# Supplementary material for: A Likelihood Approach for Real-Time Calibration of Stochastic Compartmental Epidemic Models
Source: PLoS Comput Biol. 2017 Jan 17;13(1):e1005257. doi: 10.1371/journal.pcbi.1005257 (PMC5240920; doi:10.1371/journal.pcbi.1005257)
Supplement: S1 File — (TAR.GZ) [file pcbi.1005257.s014.tar.gz › HSPH_Online-SI-Revision/PF08/n10-mild/n10-mild_PF2_table-4.pdf]

1	newly 3 weeks spec	199.	280.81	{256., 311.}	41.1106	0.14797	newly 3 weeks cumul	583.	798.04	{725., 862.}	36.8851	0.135103	newly 5 weeks spec	174.	291.79	{262., 327.}	67.6954	0.222878	newly 5 weeks cumul	973.	1378.26	{1270., 1508.}	41.6506	0.150089	newly 54 weeks cumul	2959.	4795.36	{4536., 5036.}	62.0602	0.209383
2	newly 3 weeks spec	442.	325.17	{227., 434.}	29.0023	0.158581	newly 3 weeks cumul	1121.	855.5	{592., 1097.}	27.3292	0.14632	newly 5 weeks spec	488.	374.93	{243., 524.}	26.875	0.144943	newly 5 weeks cumul	2106.	1583.54	{1083., 2100.}	27.9763	0.151019	newly 59 weeks cumul	3790.	6097.05	{4975., 6874.}	60.872	0.203235
3	newly 3 weeks spec	438.	340.52	{158., 583.}	41.0685	0.233071	newly 3 weeks cumul	1008.	853.48	{425., 1411.}	38.8968	0.205291	newly 5 weeks spec	488.	413.73	{208., 693.}	39.2725	0.209958	newly 5 weeks cumul	1997.	1647.97	{779., 2768.}	39.3455	0.213051	newly 62 weeks cumul	4433.	6181.63	{4635., 8025.}	40.5055	0.140409
4	newly 3 weeks spec	427.	299.9	{226., 413.}	30.7447	0.168162	newly 3 weeks cumul	1020.	822.84	{637., 1101.}	22.9176	0.116706	newly 5 weeks spec	527.	332.63	{249., 463.}	36.9241	0.210007	newly 5 weeks cumul	2089.	1475.68	{1132., 1989.}	30.0977	0.162982	newly 57 weeks cumul	4089.	5680.08	{5210., 6392.}	38.9112	0.141132
5	newly 3 weeks spec	105.	101.2	{84., 116.}	9.2	0.0418694	newly 3 weeks cumul	340.	331.98	{315., 351.}	3.86471	0.0171808	newly 5 weeks spec	120.	75.38	{63., 89.}	37.1833	0.206469	newly 5 weeks cumul	593.	493.91	{466., 521.}	16.7099	0.0798065	newly 37 weeks cumul	2494.	1008.59	{921., 1098.}	59.5593	0.394177
6	newly 3 weeks spec	329.	286.08	{126., 513.}	44.614	0.235458	newly 3 weeks cumul	816.	687.12	{321., 1248.}	40.9265	0.219991	newly 5 weeks spec	353.	383.2	{159., 690.}	51.2125	0.220643	newly 5 weeks cumul	1522.	1409.26	{620., 2606.}	44.2102	0.219436	newly 63 weeks cumul	3219.	6527.5	{4702., 8192.}	102.78	0.297752
7	newly 3 weeks spec	337.	296.25	{201., 431.}	25.6409	0.125722	newly 3 weeks cumul	914.	792.27	{538., 1132.}	24.7932	0.122707	newly 5 weeks spec	288.	338.71	{226., 506.}	29.1076	0.108782	newly 5 weeks cumul	1547.	1450.65	{994., 2115.}	23.989	0.110981	newly 59 weeks cumul	3151.	5766.55	{4720., 6779.}	83.007	0.258351
8	newly 3 weeks spec	276.	301.52	{264., 332.}	11.0362	0.0445741	newly 3 weeks cumul	750.	858.18	{788., 936.}	14.4587	0.0575178	newly 5 weeks spec	242.	309.81	{273., 342.}	28.0289	0.105656	newly 5 weeks cumul	1290.	1475.93	{1353., 1625.}	14.5791	0.0581507	newly 54 weeks cumul	4074.	4904.2	{4644., 5170.}	20.378	0.0801124
9	newly 3 weeks spec	372.	306.4	{216., 430.}	24.7796	0.125697	newly 3 weeks cumul	848.	825.91	{599., 1162.}	19.7866	0.0874628	newly 5 weeks spec	527.	353.62	{255., 477.}	33.4611	0.187082	newly 5 weeks cumul	1906.	1515.57	{1100., 2044.}	25.0656	0.129533	newly 58 weeks cumul	4128.	5939.14	{5255., 6761.}	43.8745	0.155865
10	newly 3 weeks spec	221.	316.11	{269., 370.}	43.0362	0.152165	newly 3 weeks cumul	630.	889.48	{765., 1039.}	41.1873	0.146956	newly 5 weeks spec	246.	327.4	{283., 380.}	33.0894	0.121729	newly 5 weeks cumul	1140.	1541.33	{1346., 1785.}	35.2044	0.128638	newly 55 weeks cumul	2746.	5193.96	{4834., 5591.}	89.1464	0.275655
11	newly 3 weeks spec	268.	285.96	{178., 415.}	25.3806	0.103582	newly 3 weeks cumul	729.	746.52	{495., 1033.}	23.6324	0.100847	newly 5 weeks spec	221.	343.49	{220., 476.}	57.5882	0.180779	newly 5 weeks cumul	1225.	1407.12	{945., 1949.}	27.5216	0.104931	newly 60 weeks cumul	2530.	5994.24	{4838., 7119.}	136.926	0.369408
12	newly 3 weeks spec	699.	208.94	{106., 365.}	70.1087	0.569329	newly 3 weeks cumul	1437.	521.8	{297., 935.}	63.6882	0.476187	newly 5 weeks spec	522.	278.2	{135., 497.}	50.2835	0.337138	newly 5 weeks cumul	2666.	1045.06	{546., 1898.}	60.8005	0.448353	newly 62 weeks cumul	3704.	6013.8	{4434., 7282.}	62.871	0.203145
13	newly 3 weeks spec	431.	291.84	{261., 322.}	32.2877	0.171	newly 3 weeks cumul	1134.	826.84	{749., 913.}	27.0864	0.138599	newly 5 weeks spec	505.	297.72	{262., 331.}	41.0455	0.231337	newly 5 weeks cumul	2205.	1423.63	{1289., 1556.}	35.4363	0.191327	newly 54 weeks cumul	4115.	4844.14	{4602., 5109.}	17.8727	0.0709745
14	newly 3 weeks spec	356.	321.99	{276., 383.}	13.4691	0.0626479	newly 3 weeks cumul	939.	896.35	{785., 1026.}	10.3951	0.0462964	newly 5 weeks spec	351.	335.65	{294., 390.}	10.584	0.0477046	newly 5 weeks cumul	1659.	1568.99	{1373., 1829.}	10.7046	0.0481987	newly 55 weeks cumul	3922.	5359.03	{4960., 5765.}	36.6402	0.134621
15	newly 3 weeks spec	198.	276.41	{216., 343.}	39.7525	0.138427	newly 3 weeks cumul	512.	750.87	{600., 951.}	46.6543	0.160078	newly 5 weeks spec	238.	306.51	{252., 403.}	29.0462	0.104972	newly 5 weeks cumul	1009.	1349.68	{1099., 1679.}	33.7661	0.120299	newly 57 weeks cumul	4011.	5482.22	{5108., 6012.}	36.6796	0.134824
16	newly 3 weeks spec	383.	269.26	{174., 369.}	32.9034	0.186327	newly 3 weeks cumul	938.	718.82	{484., 994.}	28.3753	0.152766	newly 5 weeks spec	435.	303.9	{207., 428.}	32.9241	0.185741	newly 5 weeks cumul	1811.	1308.42	{884., 1788.}	31.0127	0.171907	newly 58 weeks cumul	3950.	5557.68	{4522., 6720.}	41.0304	0.144754
17	newly 3 weeks spec	66.	272.79	{240., 301.}	313.318	0.614209	newly 3 weeks cumul	166.	759.08	{682., 841.}	357.277	0.658701	newly 5 weeks spec	111.	290.05	{257., 328.}	161.306	0.415077	newly 5 weeks cumul	390.	1333.82	{1209., 1490.}	242.005	0.532619	newly 55 weeks cumul	3319.	5084.32	{4892., 5326.}	53.1883	0.184946
18	newly 3 weeks spec	196.	291.21	{247., 353.}	48.5765	0.168138	newly 3 weeks cumul	553.	810.56	{710., 952.}	46.575	0.163273	newly 5 weeks spec	192.	309.45	{270., 361.}	61.1719	0.204468	newly 5 weeks cumul	970.	1422.02	{1255., 1631.}	46.6	0.163754	newly 55 weeks cumul	2464.	5185.06	{4857., 5536.}	110.433	0.322404
19	newly 3 weeks spec	378.	378.96	{253., 521.}	22.6138	0.0999004	newly 3 weeks cumul	1189.	1035.6	{677., 1423.}	22.8074	0.113818	newly 5 weeks spec	370.	408.06	{280., 556.}	23.0486	0.0926871	newly 5 weeks cumul	1992.	1839.11	{1253., 2468.}	21.2395	0.101141	newly 58 weeks cumul	3457.	5667.71	{4533., 6668.}	63.9488	0.209591
20	newly 3 weeks spec	289.	355.53	{212., 482.}	34.6747	0.130604	newly 3 weeks cumul	900.	940.25	{597., 1240.}	23.05	0.100258	newly 5 weeks spec	356.	402.73	{241., 541.}	29.132	0.118186	newly 5 weeks cumul	1637.	1728.73	{1083., 2274.}	24.3366	0.10528	newly 59 weeks cumul	3969.	5976.57	{4432., 7022.}	50.6271	0.172006
21	newly 3 weeks spec	420.	347.41	{227., 474.}	23.8119	0.127241	newly 3 weeks cumul	1015.	919.96	{637., 1250.}	22.0828	0.10853	newly 5 weeks spec	456.	394.08	{246., 539.}	22.1491	0.114437	newly 5 weeks cumul	1966.	1686.78	{1140., 2261.}	22.3164	0.115483	newly 59 weeks cumul	3646.	5990.57	{4811., 7056.}	64.3053	0.210575
22	newly 3 weeks spec	279.	289.37	{236., 352.}	11.81	0.0491559	newly 3 weeks cumul	788.	820.51	{701., 967.}	10.2805	0.0426363	newly 5 weeks spec	256.	308.36	{254., 370.}	21.8438	0.0829355	newly 5 weeks cumul	1343.	1431.87	{1224., 1670.}	11.0372	0.0448659	newly 55 weeks cumul	3250.	5146.07	{4774., 5494.}	58.3406	0.198874
23	newly 3 weeks spec	399.	342.36	{209., 480.}	27.5489	0.14321	newly 3 weeks cumul	1073.	876.13	{548., 1226.}	26.6887	0.143952	newly 5 weeks spec	394.	420.03	{232., 593.}	29.302	0.125788	newly 5 weeks cumul	1876.	1689.7	{1026., 2373.}	25.9403	0.12978	newly 60 weeks cumul	3098.	6628.49	{5460., 7464.}	113.96	0.327332
24	newly 3 weeks spec	199.	325.25	{257., 425.}	63.4422	0.205309	newly 3 weeks cumul	621.	900.64	{716., 1143.}	45.0306	0.154543	newly 5 weeks spec	188.	343.3	{276., 441.}	82.6064	0.254562	newly 5 weeks cumul	1038.	1582.68	{1282., 2025.}	52.474	0.17671	newly 56 weeks cumul	2085.	5399.96	{4951., 5876.}	158.991	0.410917
25	newly 3 weeks spec	253.	218.63	{107., 359.}	37.332	0.192372	newly 3 weeks cumul	550.	534.68	{274., 813.}	34.4582	0.158898	newly 5 weeks spec	366.	300.1	{141., 535.}	41.3661	0.226537	newly 5 weeks cumul	1317.	1095.27	{546., 1817.}	37.0456	0.196983	newly 63 weeks cumul	2982.	6182.74	{4294., 7839.}	107.335	0.305796
26	newly 3 weeks spec	272.	325.02	{285., 356.}	19.7132	0.076314	newly 3 weeks cumul	780.	933.35	{840., 1026.}	19.6603	0.0763661	newly 5 weeks spec	259.	329.81	{297., 380.}	27.3398	0.103287	newly 5 weeks cumul	1322.	1599.24	{1447., 1759.}	20.9713	0.0813327	newly 54 weeks cumul	2407.	5157.34	{4866., 5455.}	114.264	0.330398
27	newly 3 weeks spec	426.	302.21	{248., 378.}	29.6315	0.157364	newly 3 weeks cumul	1154.	834.95	{714., 1034.}	27.9575	0.146858	newly 5 weeks spec	412.	322.43	{269., 395.}	23.3325	0.117975	newly 5 weeks cumul	2041.	1472.06	{1267., 1791.}	28.1764	0.147886	newly 56 weeks cumul	3536.	5358.42	{4924., 5916.}	51.539	0.179479
28	newly 3 weeks spec	120.	60.54	{48., 71.}	49.55	0.302078	newly 3 weeks cumul	339.	206.74	{191., 226.}	39.0147	0.216211	newly 5 weeks spec	111.	46.1	{34., 57.}	58.4685	0.388917	newly 5 weeks cumul	576.	304.83	{280., 332.}	47.0781	0.277672	newly 34 weeks cumul	2091.	603.56	{529., 671.}	71.1353	0.541702
29	newly 3 weeks spec	361.	328.35	{223., 457.}	21.6371	0.104429	newly 3 weeks cumul	1007.	905.12	{610., 1277.}	21.3287	0.103246	newly 5 weeks spec	360.	349.21	{244., 471.}	21.5083	0.0969034	newly 5 weeks cumul	1758.	1591.7	{1103., 2148.}	21.2457	0.102115	newly 57 weeks cumul	3678.	5405.21	{4647., 6226.}	47.2656	0.164911
30	newly 3 weeks spec	367.	319.	{227., 409.}	19.9292	0.100798	newly 3 weeks cumul	970.	895.04	{619., 1124.}	18.4	0.0882626	newly 5 weeks spec	361.	333.95	{236., 445.}	19.4654	0.0923971	newly 5 weeks cumul	1701.	1565.22	{1104., 1974.}	18.6596	0.0893239	newly 56 weeks cumul	3445.	5203.29	{4282., 6049.}	51.1469	0.175316
31	newly 3 weeks spec	288.	253.52	{111., 438.}	41.5556	0.213112	newly 3 weeks cumul	670.	615.68	{292., 1017.}	38.4866	0.187406	newly 5 weeks spec	360.	347.65	{147., 614.}	43.9472	0.210888	newly 5 weeks cumul	1418.	1266.63	{565., 2127.}	40.3364	0.204323	newly 63 weeks cumul	2824.	6391.86	{4790., 8149.}	126.341	0.346005
32	newly 3 weeks spec	126.	90.17	{75., 105.}	28.4365	0.148778	newly 3 weeks cumul	395.	306.49	{277., 333.}	22.4076	0.111413	newly 5 weeks spec	145.	68.03	{54., 83.}	53.0828	0.33398	newly 5 weeks cumul	687.	452.01	{414., 491.}	34.2052	0.182901	newly 36 weeks cumul	3008.	900.04	{770., 1015.}	70.0785	0.526127
33	newly 3 weeks spec	466.	312.89	{241., 391.}	32.8562	0.180637	newly 3 weeks cumul	1168.	849.42	{671., 1047.}	27.3168	0.144954	newly 5 weeks spec	533.	353.37	{281., 443.}	33.7017	0.185385	newly 5 weeks cumul	2236.	1541.53	{1234., 1872.}	31.0586	0.167575	newly 57 weeks cumul	4145.	5890.11	{5317., 6470.}	42.1016	0.151203
34	newly 3 weeks spec	301.	308.91	{230., 395.}	17.8771	0.0751448	newly 3 weeks cumul	838.	845.75	{640., 1065.}	16.3926	0.070178	newly 5 weeks spec	335.	341.06	{267., 423.}	16.5612	0.0706518	newly 5 weeks cumul	1525.	1516.21	{1172., 1920.}	15.8498	0.0687902	newly 57 weeks cumul	3835.	5661.77	{5110., 6442.}	47.6342	0.167431
35	newly 3 weeks spec	430.	380.5	{272., 503.}	20.4326	0.0996988	newly 3 weeks cumul	1171.	1055.65	{736., 1404.}	19.3245	0.0933021	newly 5 weeks spec	358.	402.18	{292., 539.}	22.1564	0.085979	newly 5 weeks cumul	1960.	1853.99	{1338., 2438.}	18.7209	0.0863868	newly 57 weeks cumul	3634.	5682.39	{4625., 6755.}	56.3674	0.189914
36	newly 3 weeks spec	452.	328.5	{208., 457.}	29.9823	0.167557	newly 3 weeks cumul	1197.	871.8	{558., 1216.}	29.6692	0.163509	newly 5 weeks spec	398.	374.72	{249., 502.}	21.9598	0.104102	newly 5 weeks cumul	2080.	1602.81	{1048., 2181.}	26.5861	0.143013	newly 59 weeks cumul	3700.	5991.92	{4854., 6962.}	61.9438	0.204598
37	newly 3 weeks spec	333.	286.04	{134., 499.}	40.2162	0.20791	newly 3 weeks cumul	782.	712.94	{347., 1207.}	38.2839	0.185546	newly 5 weeks spec	357.	365.98	{185., 639.}	42.2017	0.185638	newly 5 weeks cumul	1618.	1411.31	{689., 2413.}	39.1959	0.19843	newly 62 weeks cumul	2716.	6273.36	{4600., 7922.}	130.978	0.355315
38	newly 3 weeks spec	369.	333.09	{226., 453.}	21.3089	0.102422	newly 3 weeks cumul	949.	894.45	{616., 1239.}	19.4089	0.0899235	newly 5 weeks spec	348.	382.06	{272., 523.}	22.8046	0.0902966	newly 5 weeks cumul	1684.	1638.66	{1149., 2206.}	19.8397	0.0891007	newly 58 weeks cumul	4183.	6074.45	{5274., 7026.}	45.295	0.159896
39	newly 3 weeks spec	526.	301.54	{173., 430.}	42.673	0.263863	newly 3 weeks cumul	1168.	792.5	{459., 1117.}	32.3476	0.189614	newly 5 weeks spec	460.	354.97	{216., 491.}	26.2065	0.146179	newly 5 weeks cumul	2156.	1478.91	{867., 2073.}	31.6851	0.184695	newly 59 weeks cumul	3091.	5844.51	{4730., 6999.}	89.0815	0.271401
40	newly 3 weeks spec	95.	50.3	{42., 60.}	47.0526	0.280422	newly 3 weeks cumul	322.	175.09	{164., 186.}	45.6242	0.265295	newly 5 weeks spec	79.	38.86	{30., 49.}	50.8101	0.315293	newly 5 weeks cumul	527.	258.76	{242., 273.}	50.8994	0.309478	newly 32 weeks cumul	1882.	497.63	{434., 556.}	73.5584	0.579598
41	newly 3 weeks spec	565.	264.04	{112., 548.}	56.5947	0.414985	newly 3 weeks cumul	1036.	643.94	{304., 1220.}	48.5869	0.303554	newly 5 weeks spec	573.	355.87	{140., 742.}	49.8866	0.324666	newly 5 weeks cumul	2224.	1317.73	{581., 2798.}	50.3988	0.328539	newly 63 weeks cumul	3132.	6388.65	{4677., 8190.}	103.98	0.300574
42	newly 3 weeks spec	232.	278.84	{241., 342.}	21.3276	0.0809808	newly 3 weeks cumul	680.	786.69	{680., 936.}	16.5544	0.06372	newly 5 weeks spec	278.	297.89	{256., 344.}	10.8813	0.0435227	newly 5 weeks cumul	1255.	1372.1	{1198., 1586.}	11.5665	0.0456677	newly 55 weeks cumul	2911.	4990.6	{4578., 5399.}	71.4394	0.23299
43	newly 3 weeks spec	534.	254.38	{142., 360.}	52.4757	0.351642	newly 3 weeks cumul	1357.	637.64	{373., 885.}	53.1408	0.351827	newly 5 weeks spec	396.	331.34	{164., 472.}	28.9444	0.155198	newly 5 weeks cumul	2311.	1261.81	{682., 1765.}	45.9589	0.29132	newly 61 weeks cumul	3391.	6312.31	{5086., 7433.}	86.1489	0.264912
44	newly 3 weeks spec	300.	299.8	{237., 362.}	13.34	0.0571378	newly 3 weeks cumul	794.	819.39	{656., 1006.}	13.4673	0.0558324	newly 5 weeks spec	309.	332.44	{271., 397.}	14.5113	0.057279	newly 5 weeks cumul	1422.	1476.05	{1187., 1821.}	13.1301	0.0537815	newly 57 weeks cumul	3738.	5697.19	{5143., 6211.}	52.4128	0.181695
45	newly 3 weeks spec	534.	272.58	{104., 547.}	52.4045	0.387346	newly 3 weeks cumul	994.	631.97	{262., 1278.}	45.9748	0.298814	newly 5 weeks spec	717.	406.48	{148., 835.}	50.0614	0.364037	newly 5 weeks cumul	2450.	1382.05	{541., 2670.}	49.2731	0.348311	newly 64 weeks cumul	4278.	6821.38	{4772., 8531.}	59.9542	0.194886
46	newly 3 weeks spec	331.	302.09	{171., 455.}	31.0423	0.147895	newly 3 weeks cumul	825.	772.93	{466., 1136.}	29.0218	0.13375	newly 5 weeks spec	370.	380.85	{204., 550.}	32.8514	0.142042	newly 5 weeks cumul	1578.	1499.55	{865., 2234.}	29.7877	0.136539	newly 61 weeks cumul	3207.	6405.57	{5033., 7406.}	99.7371	0.294944
47	newly 3 weeks spec	416.	248.65	{144., 371.}	41.2813	0.253162	newly 3 weeks cumul	828.	624.67	{398., 915.}	30.0109	0.163659	newly 5 weeks spec	448.	321.22	{185., 465.}	33.0714	0.190074	newly 5 weeks cumul	1770.	1234.85	{744., 1831.}	33.4514	0.1915	newly 61 weeks cumul	4274.	6257.82	{5168., 7415.}	46.4909	0.161281
48	newly 3 weeks spec	360.	295.92	{219., 393.}	23.8611	0.120421	newly 3 weeks cumul	945.	790.15	{602., 1043.}	22.691	0.112945	newly 5 weeks spec	310.	339.48	{256., 453.}	20.5742	0.0811402	newly 5 weeks cumul	1682.	1450.01	{1099., 1933.}	21.5345	0.105147	newly 58 weeks cumul	3197.	5892.02	{5176., 6654.}	84.2984	0.263432
49	newly 3 weeks spec	197.	306.03	{273., 347.}	55.3452	0.189277	newly 3 weeks cumul	473.	869.37	{803., 952.}	83.7992	0.263381	newly 5 weeks spec	183.	311.97	{279., 346.}	70.4754	0.229639	newly 5 weeks cumul	887.	1492.97	{1386., 1625.}	68.3168	0.225122	newly 54 weeks cumul	3344.	4986.95	{4688., 5246.}	49.1313	0.173015
50	newly 3 weeks spec	233.	330.12	{283., 371.}	41.6824	0.14874	newly 3 weeks cumul	705.	949.39	{820., 1054.}	34.6652	0.127226	newly 5 weeks spec	204.	331.43	{283., 377.}	62.4657	0.20839	newly 5 weeks cumul	1157.	1616.63	{1432., 1772.}	39.726	0.14361	newly 54 weeks cumul	2093.	5067.12	{4790., 5390.}	142.098	0.383243
